# Nanostructured Polyaniline Films Functionalized through Auxiliary Nitrogen Addition in Atmospheric Pressure Plasma Polymerization

**DOI:** 10.3390/polym15071626

**Published:** 2023-03-24

**Authors:** Jae Young Kim, Hyojun Jang, Ye Rin Lee, Kangmin Kim, Habeeb Olaitan Suleiman, Choon-Sang Park, Bhum Jae Shin, Eun Young Jung, Heung-Sik Tae

**Affiliations:** 1 School of Electronic and Electrical Engineering, College of IT Engineering, Kyungpook National University, Daegu 41566, Republic of Korea; 2 School of Electronics Engineering, College of IT Engineering, Kyungpook National University, Daegu 41566, Republic of Korea; 3 Department of Electrical Engineering, Milligan University, Johnson City, TN 37682, USA; 4 Department of Electronics Engineering, Sejong University, Seoul 05006, Republic of Korea; 5 The Institute of Electronic Technology, College of IT Engineering, Kyungpook National University, Daegu 41566, Republic of Korea

**Keywords:** atmospheric pressure plasma, nitrogen addition, plasma polymerization, polyaniline nanostructures

## Abstract

Polyaniline (PANI) was synthesized from liquid aniline, a nitrogen-containing aromatic compound, through the atmospheric pressure (AP) plasma process using a newly designed plasma jet array with wide spacing between plasma jets. To expand the area of the polymerized film, the newly proposed plasma jet array comprises three AP plasma jet devices spaced 7 mm apart in a triangular configuration and an electrodeless quartz tube capable of applying auxiliary gas in the center of the triangular plasma jets. The vaporized aniline monomer was synthesized into a PANI film using the proposed plasma array device. The effects of nitrogen gas addition on the morphological, chemical, and electrical properties of PANI films in AP argon plasma polymerization were examined. The iodine-doped PANI film was isolated from the atmosphere through encapsulation. The constant electrical resistance of the PANI film indicates that the conductive PANI film can achieve the desired resistance by controlling the atmospheric exposure time through encapsulation.

## 1. Introduction

Polyaniline (PANI), a representative conductive polymer, is a nitrogen-containing aromatic compound [1,2]. It exhibits excellent conductivity, easy manufacturability, low cost, good biocompatibility, and environmental stability and is used in various applications, such as organic electronics, anticorrosion materials, electrorheological materials, and nanobiomedicines [2,3,4,5,6]. Conventional PANI synthesis mainly involves three processes: chain growth through aniline oxidation, dimerization, and oligomerization/polymerization [6]. It requires an acidic environment and oxidants, and the resulting π-conjugated polymer has low solubility despite the wet process [6,7,8]. Although monomers with various substituent groups can be employed to improve solubility, they reduce conductivity [9].

Plasma polymerization is a synthetic method that can overcome the limitations of the existing polymerization processes. The plasma used for polymerization has a low ion temperature and a high electron temperature; this type of plasma is called non-thermodynamic-equilibrium plasma or nonthermal plasma [10,11]. Given that its nonthermal property induces high chemical activity without thermal damage, plasma polymerization is particularly advantageous for synthesizing temperature-sensitive organic polymers [11,12].

Recently, many studies have attempted to use atmosphere-generated nonthermal plasma that does not require vacuum equipment for material processing, including polymerization [13,14,15]. Atmospheric pressure (AP) plasma polymerization is attracting considerable attention as an extremely simple and eco-friendly method because AP plasma can replace chemicals or extreme synthetic environments, such as high temperatures, for the oxidation/reduction of monomers [16,17]. In particular, recent studies have demonstrated that AP plasma polymerization can produce polymer films using only electricity and monomers without the application of additional chemicals and heat [13,14,15,16]. In AP plasma polymerization, vaporized monomers are transferred into the plasma region, activated in the plasma medium, and crosslinked while leaving the plasma region [17,18].

Many studies have reported on the effect of applied power on multiple materials synthesized via AP plasma polymerization [19,20,21,22]. An important consideration for polymerization using AP plasma is the activation of monomers without breaking the chemical structure during the interaction between monomer molecules and plasma. When high applied power is used to generate a sufficient discharge in AP, the chemical structure of the monomer is weakened and an incomplete structure is synthesized because of excessive plasma energy [21,22,23]. The resulting degradation of the electrical and optical properties of the conjugated polymer eventually becomes a limitation for the functionality of the conjugated polymer as an electrode, light-emitting, and gas-sensing material in electronic applications [24,25]. AP plasma polymerization allows the precise control of the process and the resulting polymer by changing experimental parameters such as the working gas composition, plasma reactor configuration, discharge initiation/maintenance, and power source type [26,27,28,29]. In particular, the nonthermal plasma state during polymerization can be stabilized by controlling the amount of vaporized monomer molecules, the type and flow rate of additional neutral gas, and applied power. By applying additional gas to the AP plasma reactor, the plasma intensity and reactivity can be adjusted, and certain radicals, which can be expected to improve polymer properties, are also supplied.

In this study, PANI was synthesized from liquid aniline, a nitrogen-containing aromatic compound, through the AP plasma process using a newly designed plasma jet array with wide spacing between plasma jets. During PANI synthesis, an auxiliary neutral gas, such as Ar, N_2_, or O_2_, was introduced in the plasma region to control plasma intensity and generate additional radicals. The effect of the addition of N_2_, which improves the quality of the nanostructured PANI film, was examined in detail. The influences of N_2_ gas addition on the synthesis process were investigated, and the morphological, chemical, and electrical properties of the synthesized PANI were then examined.

## 2. Materials and Methods

### 2.1. AP Plasma Polymerization System

**Figure 1:**
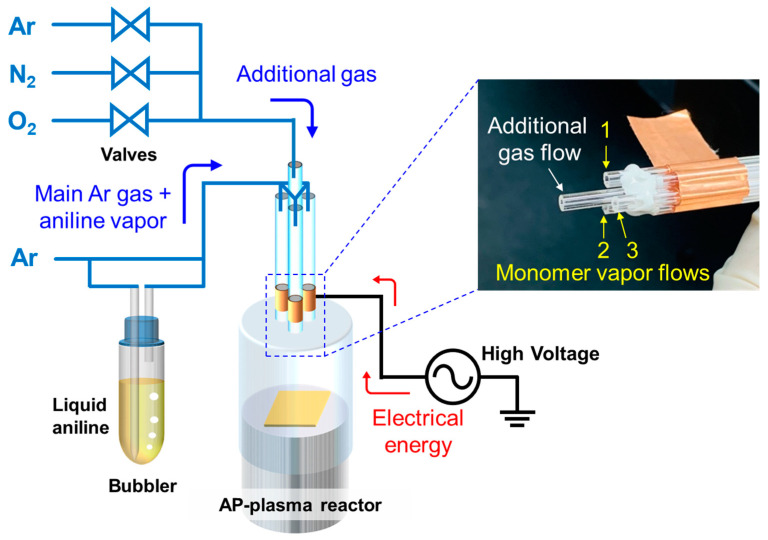
Experimental setup for the AP plasma polymerization system.

Our previous study reported on aerosol-through-plasma systems that used vaporized liquid monomers, related to AP plasma polymerization techniques, in detail [15]. Figure 1 shows the plasma polymerization equipment. The details of the experimental system, which included a monomer bubbling unit, gas feedline, driving system, and electrical monitoring instrument, are described in a previous work [15], except for the AP plasma generator. In the primary gas supply for polymerization, AP plasma was generated using Ar gas with a flow rate of 2200 sccm, and liquid aniline (MW = 93 g∙mol^−1^, Sigma-Aldrich Co., St. Louis, MO, USA) was vaporized using a bubbler and Ar gas with a flow rate of 500 sccm. The additional gas supply was split into three to test Ar, O_2_, and N_2_ gases. A sinusoidal voltage with a peak value of 10 kV and frequency of 28 kHz was then applied to the AP plasma jet (APPJ) array using an inverter-type power supply.

### 2.2. AP Plasma Reactor with Added Auxiliary Gas

The proposed AP plasma reactor comprised an APPJ array, guide tube, and substrate stand (Figure 1). The APPJ array was fabricated using four identical quartz tubes with outer and inner diameters of 3 and 1.5 mm, respectively. The APPJ array contained one tube in the center of the structure. Three other tubes were arranged in a triangle around the center tube. In the three quartz tubes arranged in a triangle, the center-to-center distance between the tubes was 7 mm. Cu tape, which was used as a powered electrode, was wrapped around three outer tubes 15 mm apart from the end of the tubes. These three tubes were then combined with each other using copper tape, thereby generating plasma in each quartz tube in the reactor. An electrodeless quartz tube was located in the center of the triangular tubes for auxiliary gas addition, and its end protruded 10 mm from the end of the outer tube to facilitate auxiliary gas supply into the polymerization area. Using the prepared plasma reactor, nanostructured PANI thin films were deposited with a 100 sccm gas flow of Ar, O_2_, and N_2_ gases for AP Ar-based plasma synthesis.

### 2.3. Electrical and Optical Characterization of Generated Plasma

A voltage probe (P6015A, Tektronix Inc., Beaverton, OR, USA) and a current probe (4100, Pearson Electronics Inc., Palo Alto, CA, USA) were used to examine APPJs during plasma polymerization. The wavelength-unresolved light emission from a photosensor amplifier (C6386-01, Hamamatsu Corp., Hamamatsu, Japan) detecting the region from the visible to the near-infrared bands were displayed on an oscilloscope (TDS3014B, Tektronix Inc., Beaverton, OR, USA). The optical emission spectra (OES) of plasma emission were acquired using a spectrometer with a fiber optic probe (USB-2000+, Ocean Optics Inc., Dunedin, FL, USA).

### 2.4. Analysis and Characterization of Nanostructured PANI Films

The shape and structure of the nanostructured polymer films were observed with a field-emission scanning electron microscope (FE-SEM; SU8220, Hitachi Korea Co., Ltd., Seoul, Korea). Based on the FE-SEM image, the pore area distribution of the film was measured through image analysis using the IMT i-Solution software (IMT i-solution Inc., Burnaby, BC, Canada).

The functional groups of PANI films synthesized by the APPJs were confirmed via attenuated total reflection Fourier transform infrared spectroscopy (ATR-FTIR; Vertex 70, Bruker, Ettlingen, Germany) at the Korea Basic Science Institute (Daegu, Korea). During the ATR-FTIR measurements, spectra ranging from 650 to 4000 cm^−1^ were obtained by averaging 128 scans.

For X-ray photoelectron spectroscopy (XPS; ESCALAB 250XI, Thermo Fisher Scientific, Waltham, MA, USA), a monochromatic Al Kα X-ray source (hυ = 1486.71 eV) operating at 15 kV and 20 mA was used to confirm surface chemical characteristics. XPS measurement was performed over an area of 500 µm × 500 µm. Wide-scan spectra were plotted with data collected every 1 eV. High-resolution measurements were recorded every 0.03 eV. Peak fitting was performed using Gaussian–Lorentzian peak shapes.

### 2.5. Iodine Doping of PANI Films for the Electrical Conductivity Test

For the electrical conductivity test, PANI films were prepared on a Si substrate with interdigitated electrodes (IDEs). Each IDE comprised 20 pairs of interdigitated and thin electrodes and had a width of 10.8 μm. The distance between IDEs was 2.54 μm. For doping halogen elements, the prepared PANI film sample and 2 g of I_2_ (Sigma-Aldrich Co., St. Louis, MO, USA, 99.99%) pellets were placed together in a Petri dish and vacuum-sealed for 30 min. When I_2_ was doped in the PANI film through sublimation, the color of the PANI film changed to dark brown.

## 3. Results and Discussion

### 3.1. Changes in Glow Discharge and PANI Films Due to Auxiliary Gas Addition during AP Plasma Polymerization

**Figure 2:**
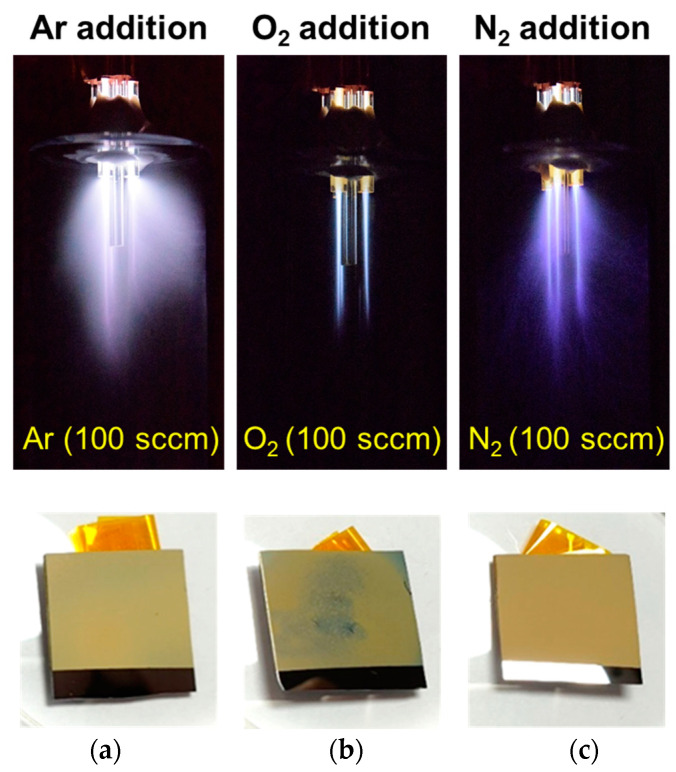
Images of glow plasma during plasma polymerization and PANI films deposited on Si substrates: image of Ar glow discharge during polymerization with the addition of (**a**) Ar, (**b**) O_2_, and (**c**) N_2_ and images of the resulting PANI films. All additional gases were used at a flow rate of 100 sccm.

Figure 2 shows the optical images of the glow discharge with the addition of auxiliary gas through the central tube of the APPJ and the resulting PANI films deposited on Si substrates. For AP plasma polymerization, Ar gas containing aniline vapor was added via the three triangular outer tubes to generate the discharge, and a nominal amount of gas was then applied to the central tube. Because each plasma jet in the triangular plasma jet array was separated from the others by a distance of 7 mm, the PANI film could be uniformly deposited on a Si substrate with dimensions of 20 mm × 20 mm without technical difficulties, as shown in Figure 2a. In this study, unlike our previous study [15], the influence of the auxiliary gas during plasma polymerization could be examined without changing the design of the guide tube or substrate stand, because an additional tube can be placed at the center of the triangular plasma jet array. Ar gas containing aniline was introduced into the plasma reactor at a flow rate of 2700 sccm, whereas the added neutral gas had a relatively low flow rate of 100 sccm. The glow emission behavior of the AP plasma and the uniformity of the resulting PANI film were extremely dependent on the type of additive gas.

When a small amount of Ar gas, which was the same as the main gas, was added, the discharge characteristics did not change considerably during plasma polymerization, and a cloudy glow emission attributed to the diffusion of aniline particles with various energy levels was observed (Figure 2a). When this cloudy glow emission was extensive and uniform inside the plasma reactor, a uniform polymer film could be obtained. As shown in Figure 2b,c, the addition of O_2_ or N_2_ during AP plasma polymerization attenuated the cloudy glow emission. The cloudy glow emissions decreased because the monomers lost energy due to collisions with neutral gases. Such collisions can be attributed to the addition of a small amount of gas different from the discharge gas to the middle of the reactor, wherein many activated monomers were located. In particular, when O_2_ was added, the cloudy glow decreased, and only plasma jets were observed. When N_2_ was added, the blurred glow emitted a blue color, indicating that some of the neutral nitrogen was transformed into excited species by the plasma energy. The plasma typically generated reactive oxygen species (ROS) and reactive nitrogen species (RNS) when O_2_ and N_2_ were added during the plasma process, respectively. However, when Ar gas, which was the same as the discharge gas, was used, the reactive byproducts caused morphological and chemical changes in PANI nanostructures.

The significant suppression of the generated plasma when a small amount of O_2_ was applied adversely affected the uniform deposition of the PANI film (Figure 2b). ROS generated by O_2_ addition not only hindered the crosslinking of the monomers but also partially etched the resulting PANI film [15]. The PANI film deposited by adding N_2_ was uniformly deposited on the Si substrate with dimensions of 20 mm × 20 mm and exhibited a matte beige appearance (Figure 2c). The images of the three PANI films in Figure 2 show that the addition of O_2_ considerably degraded the uniformity of the PANI film. Therefore, additional investigations to determine the effect of the additive gas excluded O_2_ addition.

### 3.2. Electrical and Optical Characteristics during AP Plasma Polymerization

**Figure 3:**
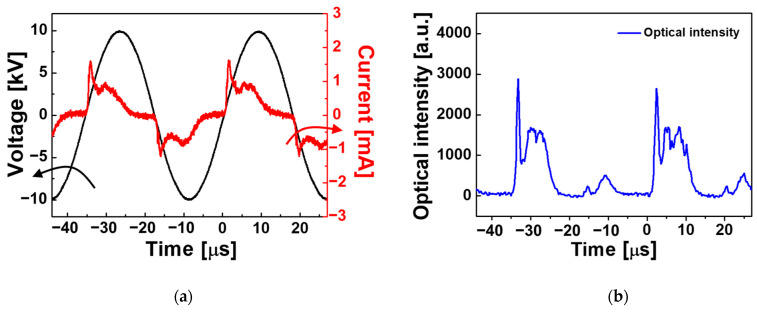
Temporal behaviors of (**a**) driving voltage (black line) and discharge current (red line), and (**b**) optical emission from the plasma generated by the proposed APPJ array.

The driving voltage, discharge current, and optical intensity were monitored, as shown in Figure 3, to examine the discharge behaviors during plasma polymerization. The driving voltage maintained a constant sinusoidal waveform with a frequency of 28 kHz and was not distorted due to electrical discharge. No change in electrical behaviors due to gas addition could be observed because the addition of a small amount of neutral gas did not fundamentally affect the discharge initiation and maintenance in the plasma reactor. Figure 3a shows the electrical behaviors monitored when Ar was added at a flow rate of 100 sccm. For removing the displacement current caused by the charging and discharging of the capacitive device, the discharge current was obtained by subtracting the current monitored when the operating voltage was applied in the absence of Ar gas from the total current monitored when plasma was normally generated. The discharge current waveform in Figure 3a shows that discharge occurred during the rising and falling periods of the voltage waveform, indicating that the discharges were successful even when the powered electrode acted not only as an anode but also as a cathode. The optical emission of the plasma jets measured near the polymerization area was periodically stable, and the optical intensity during the rising slope of the voltage waveform was higher than that during the falling slope (Figure 3b). The resulting discharge current and optical intensity demonstrated that an intense discharge was produced when the powered electrode served as the anode, demonstrating the typical behavior of dielectric barrier discharge jets generated using a single electrode without a counter electrode [30].

**Figure 4:**
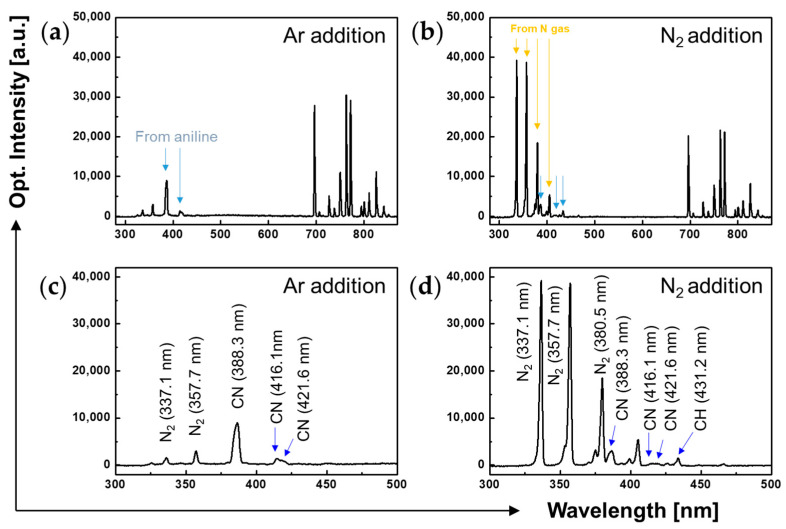
OES of plasma plumes under different gaseous conditions: OES of plasma plumes during AP plasma polymerization with (**a**) Ar addition (100 sccm) and (**b**) N_2_ addition (100 sccm). Magnified emission spectra between 300 and 500 nm in the case of (**c**) Ar addition (100 sccm) and (**d**) N_2_ addition (100 sccm).

The emission spectra of the plasma jets were obtained during AP plasma polymerization to identify the diverse reactive species created in the plasma medium. OES is a representative diagnostic method for investigating the types and energy levels of excited species in a high-pressure plasma medium and avoids perturbing the plasma medium because it does not involve the use of a diagnostic metal probe [31,32]. Figure 4 shows that emission spectra peaks between 280 and 870 nm were detected in the plasma-generating region during polymerization, demonstrating the presence of excited N_2_ as well as Ar species and carbon derivatives in the generated plasma. Many of the emission peaks in the range of 690–860 nm were attributed to the Ar discharge (Figure 4a,b), and those at 300–380 nm were primarily attributed to excited N_2_ species. Moreover, multiple carbonaceous peaks (C–N and C–H) were observed (Figure 4c,d). The addition of N_2_ gas reduced the intensity of the Ar plasma but substantially increased the N_2_ peak, indicating that some of the neutral N_2_ species produced RNS.

### 3.3. Changes in the Film Properties of PANI Nanostructures by Nitrogen Addition

**Figure 5:**
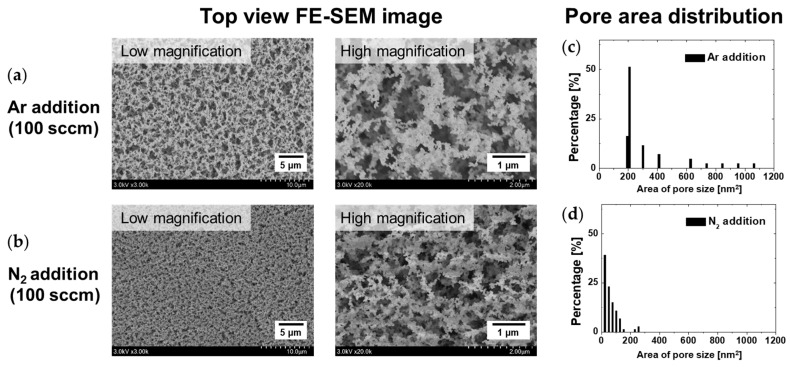
FE-SEM measurements of PANI films synthesized with the addition of (**a**) Ar and (**b**) N_2_ (top view). Pore area distribution based on the high-magnification FE-SEM images of PANI films synthesized with the addition of (**c**) Ar and (**d**) N_2_.

**Figure 6:**
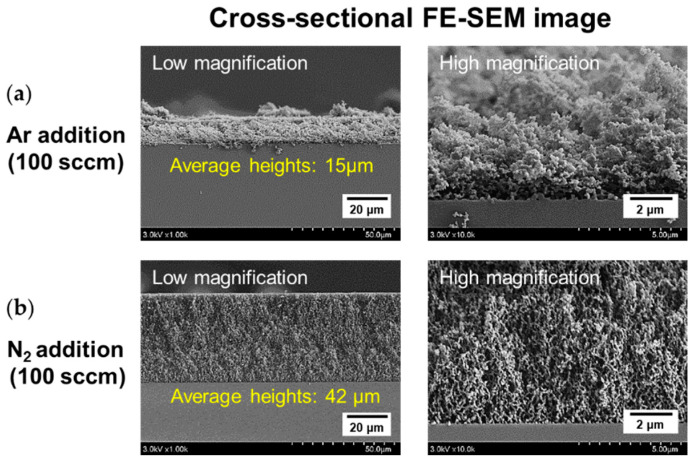
FE-SEM measurements of PANI films synthesized with the addition of (**a**) Ar and (**b**) N_2_ (cross-sectional view).

PANI films synthesized by adding Ar and N_2_ to the central tube of the proposed APPJ during AP polymerization using three triangular plasma jets were observed in detail using the FE-SEM. The PANI film deposited through the proposed AP plasma polymerization process had a porous morphology that comprised crosslinked PANI nanofibers, as shown in Figure 5a,b. Normal PANI has a coarse granular form in which nanoparticles of various sizes are synthesized and aggregated [33,34]. PANI treated with heat or additives possesses a regular and ordered structure in which nanorods and nanofibers are crosslinked [33,34,35]. During AP plasma polymerization, certain aniline-derived radicals created by plasma energy served as effective additives to promote crosslinking. The crosslinking effects on the nanostructured PANI films appear as an increase in the total pore volume and a decrease in each pore size [33]. Morphological differences were observed between the nanostructured PANI films with the addition of small amounts of Ar and N_2_. Figure 5c,d show the pore area distributions obtained by analyzing the high-magnification FE-SEM images in Figure 5a,b. The nanostructured PANI film synthesized with Ar addition had pores on the surface with an area ranging from 200 to 1200 nm^2^. However, in the case of N_2_ addition, the pore area was distributed between 20 and 300 nm^2^, which was smaller and more uniform than the film deposited by Ar gas addition. The top and cross-sectional views of FE-SEM images demonstrated that the PANI film deposited with N_2_ addition exhibited higher density and was more uniform than that deposited with Ar addition. Interestingly, the average heights of PANI films with N_2_ and Ar addition were 42 and 15 μm, respectively (Figure 6a,b). Furthermore, the PANI films demonstrated better vertical alignment with the addition of N_2_ gas. When N_2_ was added, aniline derivatives acting as additives were more effectively generated, resulting in improved crosslinking, and the thickness of the PANI film increased because of the high pore volume obtained due to crosslinking [33].

**Figure 7:**
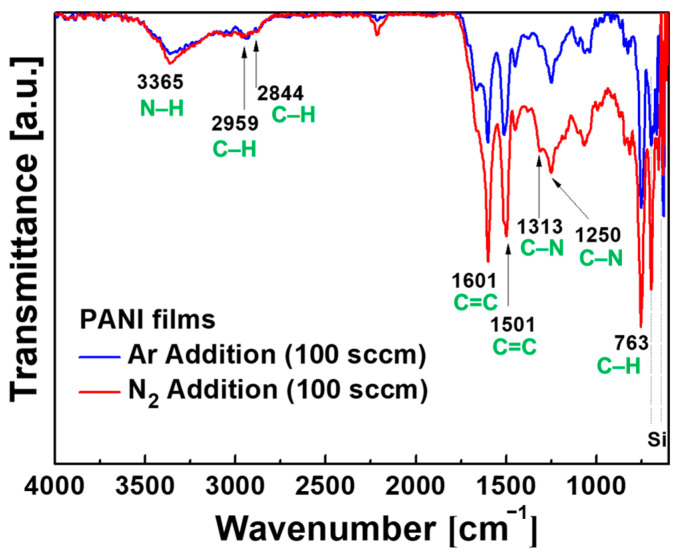
ATR-FTIR spectra of PANI films deposited with Ar and N_2_ gases.

The ATR-FTIR measurements of two PANI films deposited on Si substrates were acquired to examine the chemical characteristics of PANI films in accordance with the use of two different additive gases (Ar and N_2_; Figure 7). The ATR-FTIR spectra demonstrated the following distinctive molecular structures of PANI, which demonstrated the successful synthesis of PANI from liquid aniline: C–H deformation from the aromatic ring (763 cm^−1^), C–N stretching vibrations (1250 and 1313 cm^−1^), benzenoid stretching vibration (1501 cm^−1^), quinoid ring stretching vibration (1601 cm^−1^), C–H asymmetric stretching (2844 and 2959 cm^−1^), and N–H stretching vibration (3365 cm^−1^) [36,37]. When 100 sccm N_2_ was added during plasma polymerization, the intensity of all major peaks, including nitrogen-related peaks, increased in ATR-FTIR. Hence, the result demonstrated that the strength of the major chemical groups of PANI was enhanced because of the high nanoparticle density and uniform thickness of the nitrogen-added PANI film considering that the FTIR data were obtained with an ATR mode using the total reflection property.

**Figure 8:**
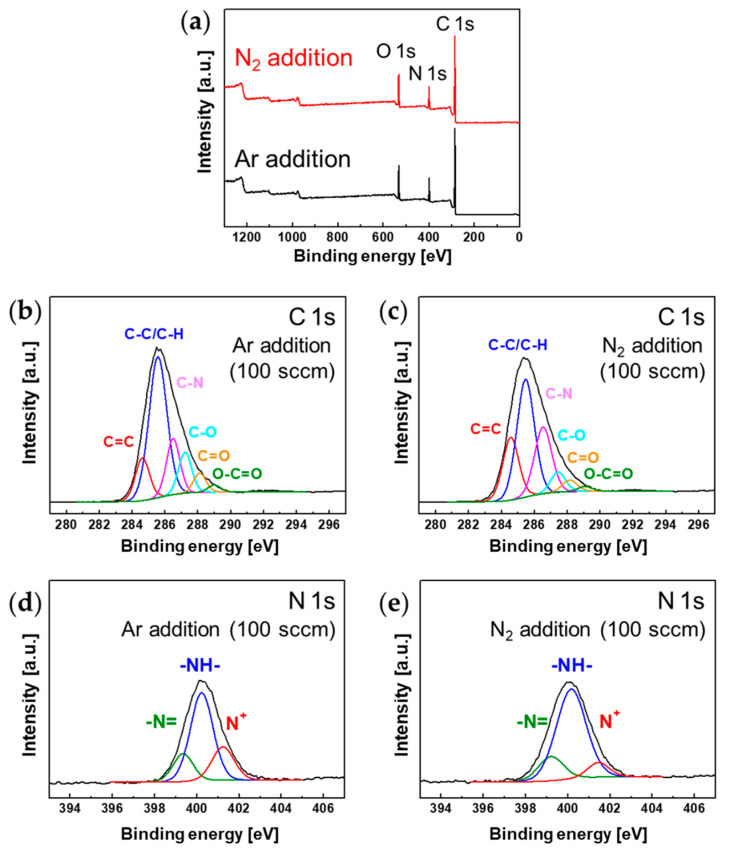
(**a**) Wide-scan XPS spectra of PANI films synthesized with additional gases (Ar and N_2_). (**b**,**c**) C 1s core-level spectra of PANI films synthesized with Ar and N_2_ gases. (**d**,**e**) N 1s core-level spectra of PANI films synthesized with Ar and N_2_ gases.

The chemical structure of the PANI film surface depending on the introduction of additional gases (Ar and N_2_) during polymerization was characterized through XPS measurements (Figure 8). Figure 8a shows the wide-scan XPS spectra of the PANI films synthesized with additional gases (Ar and N_2_). These spectra included three primary peaks assigned to oxygen (O 1s), nitrogen (N 1s), and carbon (C 1s). Table 1 summarizes the atomic composition of the PANI surface. Theoretically, a unit of PANI (C_6_H_5_N)_n_ had a C/N ratio of 6 and lacked an oxygen component. However, in the case of Ar and N_2_ addition, the surface of the synthesized PANI films exhibited similar atomic compositions, such as high amounts of oxygen and C/N ratios above 6. Inevitable exposure to water and oxygen from the air created C–O bonds on the surface of the PANI films [38]. Figure 8b–e show the high-resolution C 1s and N 1s XPS spectra of the synthesized PANI films. The C 1s profiles in Figure 8b,c were fitted to six energetic peaks at 284.6, 285.5, 286.5, 287.2, 288.1, and 289.1 eV, corresponding to the C=C, C–C/C–H, C–N, C–O, C=O, and O–C=O chemical groups, respectively [39]. The N 1s profiles in Figure 8d,e can be decomposed into three peaks centered at 399.0, 400.0, and 401.2 eV, which are assigned to –N=, –NH, and N^+^ chemical groups, respectively [38,40]. Detailed information, including chemical states and contributions, is summarized in Table 2.

Studies related to crosslinked PANI [34,40] have demonstrated that it commonly has chemical properties with fewer quinoid imine groups but more nitrogen-benzenoid groups than normal PANI [34,40,41]. In the present study, these results were observed in the form of reduced –N= groups and increased C–N groups in the XPS results provided in Table 2, indicating that the crosslinking degree of the PANI film synthesized by adding N_2_ gas was better than that of the PANI film synthesized by adding Ar. Furthermore, this result demonstrated good agreement with the increase in the number of specific aniline derivatives to promote crosslinking, as shown by FE-SEM measurements. The ratio of quinoid imine and benzenoid amine (–N=)/(–NH–) represents the redox state of PANI [42,43]. A ratio close to 0 indicates that PANI in the reduction state is dominant [40,42]. The conductivity of PANI can be improved through the oxidation of an amine (–NH–) with a dopant, such as I_2_ [44,45]. When PANI is clearly in the reduced state or the value of (–N=)/(–NH–) is low, improved electrical properties can be expected after I_2_ doping [44,45]. Therefore, as shown in Table 2, the electrical properties of the PANI film synthesized with the addition of N_2_ can be expected to improve after doping.

### 3.4. Electrical Properties of Conductive PANI Films

Because conjugated nanostructured polymer films are fabricated for use as electronic devices, they must have electrical properties. A facile method for imparting conductivity to conjugated polymers is to dope the polymers with halogen elements [44,46]. Among halogen elements, I_2_ is popular for the conductive functionalization of conjugated polymers because it is relatively easy to handle [47,48]. Therefore, I_2_ was doped into PANI films fabricated with the addition of different auxiliary gases to examine the electrical properties of the prepared PANI films.

**Figure 9:**
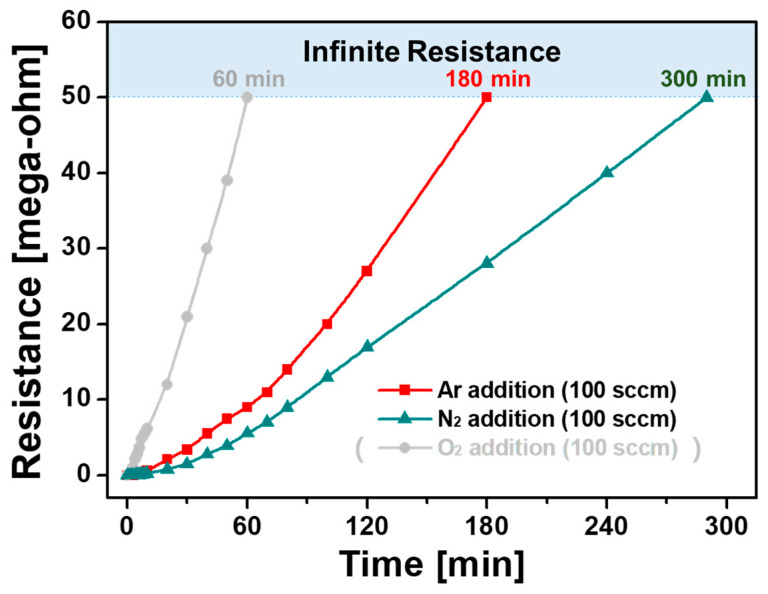
Electrical resistance of I_2_-doped PANI films prepared with different additional gases.

Figure 9 shows the resistance change of I_2_-doped PANI films synthesized with the addition of Ar and N_2_ in accordance with the exposure time to the ambient air. For a detailed comparison of the effect of gas addition, the resistance change of the I_2_-doped PANI film synthesized by adding O_2_ was recorded on the graph. The resistance measurement limit was 50 MΩ. If this limit is exceeded, the resistance is considered to be infinite. When an Ar flow of 100 sccm was added to the polymerization process, the resistance of the conductive PANI film continuously increased in the air and reached the measurement limit of 50 MΩ in 3 h. When N_2_ was added, the resistance increased more slowly and reached 50 MΩ in 5 h. The I_2_-doped PANI film obtained with the addition of N_2_ was less affected by atmospheric hydration and had better resistance stability.

This significant difference in resistance stability is closely related to the density and regularity of the PANI nanocomposite, as shown in the FE-SEM images (Figure 5 and Figure 6). The PANI nanostructured film prepared by adding N_2_ had excellent vertical orientation such that its nanostructure was extremely dense and uniform and was less affected by hydration in the ambient air, thereby improving its resistance stability. However, the PANI film prepared by adding O_2_ was significantly affected by atmospheric hydration because it was irregular even when viewed with the naked eye; moreover, its resistance stability inevitably deteriorated. Furthermore, the resistance measurement confirmed that the conductive PANI film synthesized by adding N_2_ exhibited better electrical properties than that synthesized by adding Ar, as predicted based on the XPS results.

**Figure 10:**
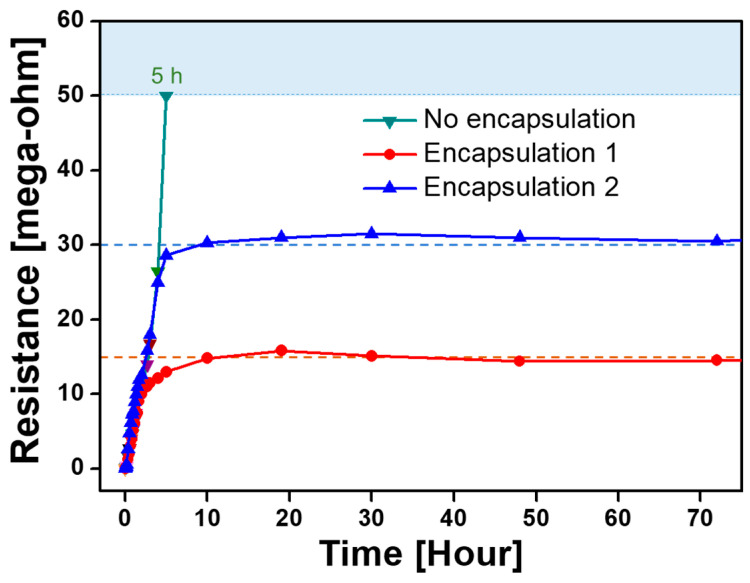
Changes in the electrical resistance of I_2_-doped PANI films subjected to different encapsulation processes over three days. Encapsulation process examined with PANI films polymerized with N_2_ gas added only.

I_2_-doped PANI films are expected to possess excellent electrical resistance stability when isolated from moisture and oxygen in the external environment by sealing. Thus, the fabricated PANI film was encapsulated with sealing tape and a film to secure stable electrical properties. The encapsulation test was performed using the PANI film polymerized with the addition of 100 sccm N_2_. This film had the best conductive performance. The change in resistance was measured over several days (Figure 10) to examine the resistance behavior of the encapsulated I_2_-doped PANI film. When the I_2_-doped PANI film was left in the atmosphere for 80 min after doping and the electrical resistance reached 10 MΩ, the PANI film on the IDE area was sealed by using polyimide tape (Kapton^®^ tape, DuPont, Wilmington, DE, USA) and an elastic sealing film (PARAFILM^®^ M, Bemis Company, Neenah, WI, USA) as well as isolated from the outside. After encapsulation, the increase in the electrical resistance of the PANI film was greatly attenuated, and the electrical resistance finally reached saturation at 15 MΩ after doping for 10 h (Encapsulation 1; red line in Figure 10). In another encapsulation test, the PANI film was encapsulated when the electrical resistance was 22 MΩ at 150 min. The resistance reached saturation at 30 MΩ 10 h after doping (Encapsulation 2; blue line in Figure 10). Monitoring the different resistances of the two encapsulated PANI films over three days revealed that both films had extremely consistent resistance values without even a change of 1 MΩ. The results indicated that the electrical resistance of the conductive PANI film can be manipulated by actively controlling the atmospheric exposure time of the polymer film via encapsulation. The long-term monitoring of the resistance of the PANI films with encapsulation revealed that three weeks were required to reach the measurement limit of 50 MΩ. The change in the resistance value of the PANI film is expected to be permanently avoided if encapsulation technology that is more advanced than the use of polyimide tape and sealing film can be employed.

## 4. Conclusions

In this study, changes in the morphological, chemical, and electrical properties of nanostructured PANI films with the addition of neutral gas for AP plasma polymerization were investigated. A separate gas tube was added to the center of the APPJ array device, and an auxiliary gas could be injected into the middle of the generated plasma. Adding a small amount of N_2_ for AP plasma polymerization not only improved the density and uniformity of the resulting nanostructured PANI film but also increased the growth rate of the PANI film. The latter effect is beneficial for rapid polymerization. Moreover, in contrast to the addition of Ar, the addition of a small amount of N_2_ could improve the resistance stability of the conductive PANI film. This property can be exploited to obtain constant electrical conductivity by isolating the conductive polymer film from moisture and oxygen in the ambient air via encapsulation. Adding a small amount of N_2_ gas was experimentally demonstrated to be a facile approach for improving the properties of conjugated polymer films without considerably affecting the maintenance of AP plasma undergoing polymerization and deposition simultaneously. Further careful investigations are required to understand exactly how nitrogen molecules/radicals increase the deposition rate of PANI films or the density of PANI nanoparticles in the film. N_2_ addition during plasma polymerization can be a practical approach for easily controlling the surface roughness of conductive polymers, thereby improving the capture of gas molecules when conductive polymers are used as the detection layer of gas sensors.

## Figures and Tables

**Table 1:** Surface atomic composition ratios of PANI films synthesized with additional gases (Ar and N_2_).

Conditions	C 1s (%)	N 1s (%)	O 1s (%)	C/N
Ar addition	76.8	11.3	11.9	6.80
N_2_ addition	76.4	11.9	11.7	6.42

**Table 2:** Summary of the contribution of each group in Figure 8b–e.

Group		Binding Energy (eV)	Composition (%)	
			Ar Addition	N_2_ Addition
C 1s	C=C	284.6	12.8	21.3
	C–C/C–H	285.5	52.7	43.0
	C–N	286.5	15.9	24.1
	C–O	287.2	11.5	6.4
	C=O	288.1	5.2	3.7
	O–C=O	289.1	1.9	1.5
N 1s	–N=	399.0	17.2	14.4
	–NH–	400.0	60.1	79.7
	N^+^	401.2	22.7	5.9
	(–N=)/(–NH–)	-	0.29	0.18

## Data Availability

Not applicable.
